# Household factors influencing cockroach infestations and helminth parasites: Insights from a rural community in Guatemala

**DOI:** 10.1371/journal.pone.0340314

**Published:** 2026-01-09

**Authors:** Wendy C. Hernández-Mazariegos, Felipe I. Torres, Manuel Rodríguez, Christian M. Ibáñez, Luis E. Escobar, Federico J. Villatoro

**Affiliations:** 1 Maestria en Ciencia Animal, Facultad de Medicina Veterinaria y Zootecnia, Universidad de San Carlos de Guatemala, Guatemala; 2 Programa de Doctorado en Medicina de la Conservación, Facultad de Ciencias de la Vida, Universidad Andres Bello, Santiago, Chile; 3 Programa de Doctorado en Ciencias Mención Biodiversidad y Biorecursos, Universidad Católica de la Santísima Concepción, Bío bío, Chile; 4 Instituto Milenio en Socio-Ecología Costera (SECOS), Santiago, Chile; 5 Unidad de Parasitología, Facultad de Medicina de Medicina Veterinaria, Universidad de San Carlos de Guatemala, Guatemala; 6 One Health Institute, Faculty of Life Sciences, Universidad Andres Bello, Santiago, Chile; 7 Department of Fish and Wildlife Conservation, Virginia Tech, Blacksburg, Virginia, United States of America; 8 Instituto de Investigación en Ciencia Animal y Ecosalud, Escuela de Estudios de Postgrado, Universidad de San Carlos de Guatemala, Guatemala; University of Uyo, NIGERIA

## Abstract

Cockroaches are vectors of pathogens and parasites that pose public health risks, especially in developing countries with poor hygiene and inadequate infrastructure. This study aimed to identify the household factors associated with the occurrence of cockroaches and the helminth parasites they carry in a rural community. Data on household infrastructure, presence of domestic animals, and insect control methods were collected from 70 households in rural Guatemala. Cockroaches were captured using traps and manually. A Generalized Linear Mixed Model revealed that households with concrete roofs had 94% lower abundance of cockroaches than those with metal sheet roofs, while the presence of cats increased cockroach abundance by 2.6 times (p < 0.05). Six genera of helminths, including the acanthocephalan zoonotic parasite *Moniliformis moniliformis*, were identified, marking the first report of such parasites in household cockroaches in Guatemala. These results highlight the need for improved housing infrastructure and integrated pest management strategies to mitigate the risks associated with cockroach-borne parasites in vulnerable communities.

## Introduction

Cockroaches are insects with a high diversity comprising over 3500 species distributed worldwide [1–3]. The most common and widespread cockroach species include the American cockroach (*Periplaneta americana*), the German cockroaches (*Blatella germanica*), and the Oriental cockroach (*Blatta orientalis*) [1,2,4]. Cockroaches are found in different wild habitats (e.g., forests, steppes, deserts), but are also commonly present in household settings or human habitats, particularly in dark rooms and kitchens, where they coexist with humans and domestic animals [2,5–7]. In household settings the presence of cockroaches has been linked to food availability and poor hygiene practices [8].

Domiciliary cockroach species have been described as gregarios [3,9]. These cockroaches shelter from the light in groups, and forage at night in search of food and water [10]. Cockroaches are associated with food, sewers, cesspools, latrines, garbage cans, chicken houses, animal cages and anywhere else there are biological waste products [3,9,11]. However, environments with inadequate sanitation facilitate cockroach infestation as the necessary food, water and harborage resources are abundant and readily accessible, compared to a clean environment [8]. This could increase the transmission of diseases due to the many pathogens they can carry [7,8].

Cockroaches are hosts and mechanical vectors of a plethora of zoonotic pathogens, like viruses (e.g., SARS-Cov-2) [12], bacteria (e.g., *Escherichia coli, Mycobacterium* spp*., Salmonella* spp.), protozoa (e.g., as *Toxoplasma gondii* and *Giardia*), fungi (e.g., *Aspergillus* spp. and *Candida*) [1,2,4,13,14], as well as they are hosts of helminth parasites (e.g., cestodes, nematodes, acanthocephalans) [15,16].

Several studies have demonstrated the presence of helminths in cockroaches, which are important in veterinary science and public health (*Hymenolepis* spp., *Taenia* sp., *Ascari* sp., *Ancylostoma* spp., *Aelurostrongylys abstrusus*, and *Moniliformis moniliformis*) [1,17,18]. Mixed parasite infestations are commonly reported in cockroaches, which could explain the non-specificity of their role as parasite vectors, so their potential to transmit diseases should not be ignored [19,20]. In addition, it should be emphasized that the presence and abundance of cockroaches is closely associated with inadequate hygiene practices [8], which remain an important public health challenge in many developing regions.

In Guatemala, there is circulation of cockroaches-borne helminth parasites in human and/or animal populations (e.g., *Hymenolepis, Moniliformis*) [21–24]. Nevertheless, cockroaches-borne parasites may be underdiagnosed in the country due their morphological similarity to other parasites, the similarity in symptoms with other parasitic diseases, and the lack of studies with this approach [13,24]. In general, the factors explaining the presence of these insects in homes, and the role they play in maintaining parasitic cycles, are not well understood [8]. In addition to contaminating food and carrying many microorganisms, cockroaches can cause economic losses to residents as a result of pest control efforts or the loss of contaminated food [8]. Investigating the environmental conditions in communities where cockroaches thrive, and the microorganisms they carry, could help fill the knowledge gaps in cockroach management programs.

Despite the global importance of understanding the factors contributing to the spread of parasites through cockroaches, there is a significant knowledge gap in developing countries like Guatemala. In developing countries poverty and poor environmental hygiene practices have been noted as predisposing factors to illness transmitted by insects such as cockroaches [25–27]. Helminth parasites constitute the second largest group of organisms pathogenic to vertebrates which are transmitted by cockroaches [2,13]. However, there is a lack of studies addressing the factors associated with the presence of cockroaches and the parasites they harbor in these regions.

This study aims to help bridge this gap by evaluating household factors associated with the presence of cockroaches and identifying the helminth parasites they carry in a rural community in Guatemala. To achieve this, information was gathered on infrastructure, the presence of animals, and any use of cockroach control, with cockroaches being captured through traps and manual collections.

## Methods

### Study area

**Fig 1:**
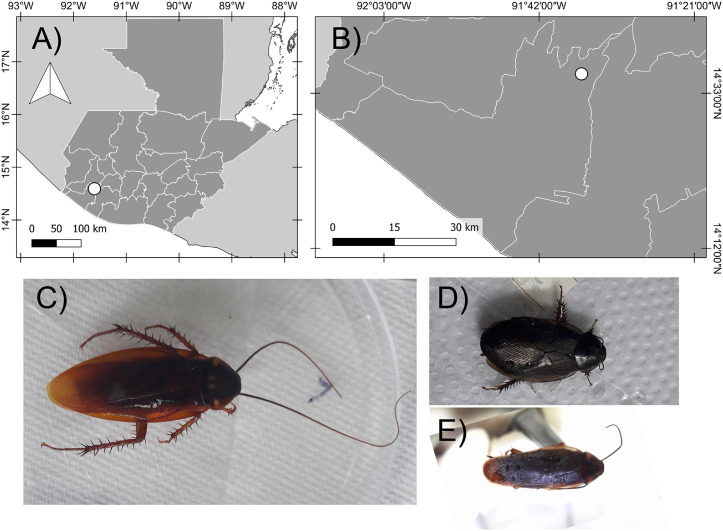
Geographic location of the study site and adult cockroach specimens identified in the study. (A) Map showing Guatemala (dark gray) and the study site location (white dot). (B). Map showing the study site location in the Retalhuleu department, Guatemala. (C) *Periplaneta americana* adult specimen. (D) *Blatta orientalis* adult specimen. (E) *Blattella germanica* adult specimen.

This study was conducted from May to June 2018 in The Caserío Santa Teresita, San Martín Zapotitlán, Retalhuleu, a rural area located in occidental south Guatemala (14°35’42.39”N, 91°35’47”W) (Fig 1), with an extension area of ~ 30,000 m^2^. The maximum temperature in this locality is 34^°^C, with a minimum of 22^°^C, and relative humidity of up to 71% [28,29]. The population size is about 400 people (~5.08 people per household), the average salary in the sector is the lowest in the country (monthly salary was ~ GTQ 2,460/ USD $ 320 in 2018), with a general poverty index of 52.7%. The municipality of San Martín Zapotitlán does not have any registered protected areas, and does not restrict land use in any specific area [30,31]. The streets in the study area vary between dirt and paved (concrete) roads. Localities farther from the municipal capital, such as Caserío Santa Teresita, require particular attention regarding basic services like access to drinking water, drainage, and waste collection [30,31].

This site was selected for this study based on convenience criteria, including prior knowledge of the community, accessibility, and reported presence of cockroaches in households [W.H. personal observation, 2018]. Although there are no official reports of helminth infections transmitted by cockroaches for this area, the second most important morbidity causes in the Retalhuleu department are parasitic illnesses [32]. However, the presence of domestic cockroaches is common in the area, and their potential role as mechanical vectors of parasites has not been investigated locally prior to this study.

### Data collection and cockroach capture

From the 94 households in the locality, we sampled 70 of them. The smallest sample size was calculated based on the total households at the study site (N = 94), with a confidence interval of 95%, an assumed prevalence of 50% and an error of 0.1%. We used a convenience-based approach, visiting households in sequence and requesting participation. Of the total households, 70 provided consent and were included in the study. In each household, data were collected on factors such as the construction material used for floors, walls and roofs, the presence of domestic animals, and methods of cockroach control. Insects were captured manually and with the use of traps [33]. Manual capture was performed using disposable gloves to avoid direct contact with specimens. Cockroaches were collected directly by hand from location withing the household where people reported high infestation levels, including furniture, drawers, cabinets, containers, and other sheltered microhabitats. Captured individuals were immediately placed into sterile containers. Traps (15 cm high x 7.5 cm wide) were built with recycled plastic bottles of 2000 ml, which were cut in half and had the top inverted as a funnel into the bottom. Traps were coated with a thin layer of Vaseline® to prevent the insects from escaping, and bread and cookies were placed inside the traps as bait, following previous capture methods [33–35].

Traps were placed inside and around the household where people reported cockroaches, where they believed the insects were present, or where they gave us permission to place them. For example, traps were placed in kitchens, bedrooms, living rooms, backyards or near sewage areas. Three traps were installed per household for two nights, between 15:00 and 18:00, and checked the following day at 8:00 hours.

The captured cockroaches were transported to a workstation, where each insect was euthanized by decapitation in a petri dish [17,19,36]. The insects were identified to species level and preserved in separate vials containing formol 2%. Species identification was performed based on morphological characteristics such as size and color, following morphological description by [2]. The age (adults and nymphs [juveniles]) was categorized by the presence of wings (present in adults only) and sex (females and males) was determined by the presence or absence of stylets on the posterior of the abdomen (only males have stylets), following [2] and [3]. Informed verbal and written consent were obtained from the household owner before conducting data collection and cockroaches trapping procedures.

### Laboratory methods

#### Sample processing and parasitology examination.

Cockroaches were analyzed and processed at the Laboratory of the Parasitology, Faculty of Veterinary Medicine, University of San Carlos of Guatemala. Each insect was dissected in two sections, including the external area (head, wings, and legs) and the internal area (intestines and hemocoel) [15]. Both areas were processed to search for parasite and parasite eggs using a sedimentation technique. The dissected parts were macerated in 9 ml of 0.9% saline solution, sieved and centrifuged at 2500 rpm for 5 min. The resulting sediment was stained with Lugol’s iodine solution (2%), and examined for helminths eggs using a Leica® light microscope at 10x and 40x magnification. Helminth parasites were identified using diagnostic guides and published studies about morphological characteristics of pinworms and egg stages [37–42].

### Data analysis

Household and cockroach data were summarized using descriptive statistics. A Generalized Linear Mixed Model (GLMM) [43] was used to determine the associated with the quantity of *P. americana* by factor, considering floor type, wall type, roof type, insect control method and presence of animals as predictors (fixed effects), and household (ID) and capture method (trap or manual) as random effects. Random effects were included to examine the variability of houses condition and method traps efficacy. Although the optimizer algorithm BOBYQCA was used to improve performance in non-linear mixed models, it is more stable of strong correlations between random effects, and helps to better converge the model [44]. To determine statistical significance of fixed effects an alpha level of 0.05 throughout all stages of statistical modeling. Fixed effects were considered statistically significant when p-values were below 0.05. GLMM was used to simultaneously examine many possible determinant presences of cockroaches. Doing so allowed us to assess each factor’s impact while controlling for the effects of all others [43]. Delta AICc method (Akaike Information Criteria) was used to compare each model with the general “best predictor” model [45]. Also, a Generalized Variance Inflation Factor (GVIF) analysis was carried out to evaluate the collinearity between the factors.

Additionally, the prevalence of each parasite species was calculated as the number of *P. americana* individuals infected with that parasite divided by the total number of cockroaches examined in each category, following [46]. Also, prevalence of parasitized cockroaches by sex and stage was calculated, and the comparison of the number of parasitized cockroaches by sex and stage were analyzed with Chi-square and Fisher’s Test. Finally, prevalence of parasitized cockroaches by location capture (e.g., kitchen, bedroom, sewer) were calculated [46]. We recognize that in rural homes, structural separation between rooms may be limited, allowing cockroaches to freely circulate [5]. Therefore, the classification of capture sites reflects the functional use of space as reported by residents and observed during trap placement, rather than strict architectural separation. The observed differences by capture site should be interpreted as general trends, rather than strict spatial associations. All data analysis was conducted using the R software v4.4.1 [47], statistical details are available in https://github.com/wendychm7/Cockroaches-analysis.

## Results

### Factors associated with the presence of cockroaches

The 70 households studied had different construction materials for flooring, including concrete (industrial cement mix and gravel n = 44), dirt floor (bare soil without concrete or tile covering, n = 11), and a combination of concrete and dirt floor (n = 15). Construction materials for walls varied from concrete blocks (or cinder blocks, n = 40), corrugated metal sheets (aluminum or galvanized steel, n = 5), wooden planks (irregular or matched boards, sometimes reclaimed, n = 2), plastic sheets (nylon or polyethylene film, n = 1), blocks combined with other materials (n = 17), metal sheets combined with other materials (n = 3), and wooden planks combined with other materials (n = 2). Roof type included corrugated metal sheets (n = 68) and concrete (n = 2) (S1 Table).

Additionally, households had the presence of diverse domestic animals, including dogs (n = 34), cats (n = 27), and poultry (n = 26). Most households had some type of cockroach control method (implemented = 49, not implemented = 21) (S1 Table).

A total of 260 cockroaches were found in 38 (54%) households, with a range of 1–32 in household abundance. From the 260 cockroaches captured 258 were identified as *Periplaneta americana*, one as *Blattella germanica*, and one as *Blatta orientalis* (Fig 1)*.* The highest number of cockroaches were found in kitchens (220), followed by bedrooms (14), backyards (11), laundry areas (6), living rooms (5), and sewers (4) (S2 Table).

The type of roofing material and presence of cats were the strongest predictors of cockroach abundance (Table 1, S4 Table). Regarding GVIF results, no variable presented collinearity according to the adjusted thresholds (S3 Table). However, the wall material variable was excluded from the final GLMM analysis given the results during the selection process based on the Delta AICc criteria (S4 Table). The species *B. germanica* and *B. orientalis* were excluded from the analyses due to their low number and because no parasites were recovered from them.

**Table 1:** Generalized Linear Mixed Model analyses of household factors influencing cockroach abundance in a rural community in Guatemala. Delta AIC (ΔAIC) is the decrease in the Akaike’s information criterion (AIC) for the full model without that variable.

Factor	Estimate	Std. Error	z value	p value	Odds Ratio	95% confidence interval		ΔAIC
						Lower	Upper	
Floor material								
Concrete	Reference							2.53
Dirt	0.595	0.435	1.367	0.171	1.813	0.772	4.256	
Concrete-Dirt	0.005	0.424	0.013	0.989	1.005	0.437	2.312	
Roof material								11.88
Metal sheet	Reference							
Concrete	-2.598	1.294	-2.007	0.044*	0.074	0.006	0.941	
Cat presence								11.57
No	Reference							
Yes	0.966	0.342	2.824	0.004*	2.629	1.344	5.142	
Dog presence								6.2
No	Reference							
Yes	0.159	0.330	0.484	0.628	1.173	0.614	2.241	
Poultry presence								9.45
No	Reference							
Yes	0.264	0.345	0.766	0.443	1.303	0.662	2.564	
Control method								6.47
No	Reference							
Yes	-0.394	0.360	-1.094	0.2737	0.647	0.332	1.336	

Asterisks indicate significance (p < 0.05).

### Helminth parasites and their prevalence in cockroaches

**Fig 2:**
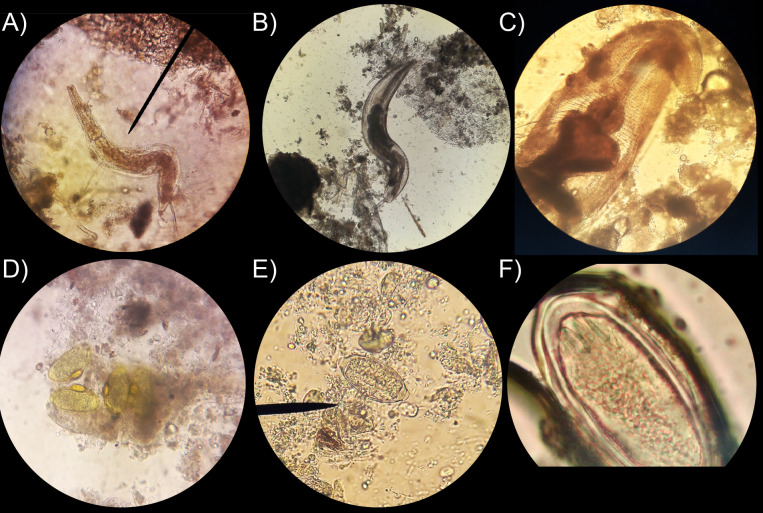
Parasites identified in cockroach specimens under a stereoscopic microscope. Microscopic view, objective 40X. (A) *Hammerschmidtiella* sp. adult. (B) *Leidynema appendiculatum* adult. (C) Anterior section of *Thelastoma* sp. adult (D) Detail of *Protrellus* eggs within a dissected specimen. (E) Egg of *Trichuris* sp. (F) Egg of *Moniliformis moniliformis* showing three characteristic hooks at the top left. Identification was based on diagnostic morphological characteristics following taxonomic references; no morphometric measurements were performed.

Six species of helminths were identified, including five nematodes and one species of acanthocephalan (Fig 2, Table 2). Helminths included *Hammerschmidtiella* sp., found in 26% (66/258) of the cockroaches, followed of *Leidynema appendiculatum* in 3.1% (8/258), *Thelastoma* sp. in 2.7% (7/258), *Protrellus* sp. in 0.8% (2/258), and *Trichuris* sp*.* in 0.4% (1/258) of the cockroaches. The acanthocephalan recorded was *Moniliformis moniliformis*, found in 0.8% (2/258) of the cockroaches. Additionally, taenia-type-eggs were observed in 1.2% (3/258) of cockroaches.

**Table 2:** Abundance and prevalence of helminth parasites identified in *Periplaneta americana* from a rural Guatemalan community.

*Periplaneta americana*			*Hammerschmidtiella* sp.	*Leidynema appendiculatum*	*Thelastoma* sp.	*Protrellus* sp.	*Trichuris* sp.		*Moniliformis* moniliformis	Taenia- type egg
Variable	level	n	n (%)	n (%)	n (%)	n (%)	n (%)	n (%)		n (%)
Sex	Female	69	21 (30)	3 (4.3)	4 (5.7)	0	1 (1.4)	0		1 (1.4)
	Male	33	7 (21)	0	0	1 (1.4)	0	0		0
Stage	Adult	102	28 (27)	3 (2.9)	4 (3.9)	1 (0.9)	1 (0.9)	0		1 (0.9)
	Nymph	156	38 (25)	5 (3.2)	3 (1.9)	1 (0.6)	0	2 (1.3)		2 (1.3)

Variable: classification of *P. americana* by sex (female, males) and development stage (adult, nymph).

Level: categories within each variable.

n: number of *P. americana* examined in each category, and number (percentage = prevalence) of cockroaches parasitized by each helminth taxon.

Pinworms and egg helminths were identified based on their diagnostic morphological traits, following published taxonomic guides and references studies [37–42]. Pinworm nematodes were recognized by the presence of swollen middle esophagus or pseudobulb. *Hammerschmidtiella* was recognized by the presence of a swollen middle esophagus or “pseudobulb” (Fig 2A), characteristic that distinguished it from *Thelastoma* sp. [38–40]. In contrast, *Leidynema* was identified by the presence of a gastric cecum, a blind appendix extending from the anterior intestine just posterior to the end bulb of the esophagus [38,41] (Fig 2B). *Thelastoma* species were distinguished from other thelastomatid pinworms by their distinctive esophageal structures [38,42] (Fig 2C). *Protrellus* eggs are oval and exhibit a lateral circular ridge on the shell [38] (Fig 2D), whereas *Trichuris* eggs are barrel-shaped, thick-shelled, and have a pair of “plugs” at each end [37] (Fig 2E). *Moniliformis* eggs are elongated-oval, with a thick transparent shell, and a larva (acanthors) with rostellar hooks [37] (Fig 2F). A summary table highlighting the main diagnostic differences among these taxa is provided as S5 Table.

The prevalence of parasitized cockroaches varied among sex and stage (Table 2). Overall, females appear to have the highest parasitism rates 37.7% (26/69), especially for *Hammerschmidtiella* sp*.*, while males generally had lower parasite prevalence 21.2% (7/33) across species (S6 Table). By stage, 32.35% (33/102) of nymphs (juveniles) and 31.4% (49/156) of adults were parasitized (S6 Table). No differences were found in the prevalence of parasitized cockroaches by sex and sage (Chi-squared, p ≥ 0.05; Fisher’s Test, p ≥ 0.05).

### Parasitized cockroaches by capture location

From the total *P. americana* captured 32% (82/258) were parasitized by at least one of the six identified helminth species (S6 Table). Parasitized cockroaches were found in 27 (39%) out of the 70 households inspected. The prevalence of parasitized cockroaches by capture location was 75% (3/4) in sewers, followed by bedrooms at 47% (8/17), kitchens at 30.5% (66/216), backyards at 30% (3/10), living rooms at 20% (1/5), and laundry rooms at 17% (1/6).

Besides the aforementioned parasites, we found six genera of protozoa, including *Eimeria* sp., *Cyclospora* sp., *Cystoisospora* sp., *Entamoeba* sp., *Balantidium* sp., and *Nyctotherus* sp.

## Discussion

This study detected factors associated with the presence of cockroaches and their parasites, specifically in *Periplaneta americana*, in a rural community in Guatemala. Other studies have recorded the presence of cockroaches parasites in Guatemala, without considering the factors associated with their presence [14,48]. Cockroaches, particularly *P. americana*, pose significant public health risks in households, especially in developing countries [16,33,36,49,50]. Cockroaches are associated with the transmission of foodborne diseases like diarrhea, dysentery, and typhoid fever [3,13]. Cockroaches’ presence in kitchens indicates poor food hygiene and can lead to serious health consequences [51].

### Factors associated with cockroaches

Households with concrete roofs exhibited a significantly lower cockroach abundance, with concrete construction associated with a 94% reduction in the number of cockroaches compared to homes with metal roofs. In contrast, the presence of cats was positively associated with cockroach abundance, as households reporting cats harbored approximately 2.6 times more cockroaches than those without (Table 1).

Following our results, the type of roof (i.e., concrete) and presence of domestic animals (i.e., cats) are the best predictor factors to the abundance of cockroaches of *P. americana* (Table 1). These results are consistent with previous studies which report association between roach infestation and poor building quality (e.g., rotting wood, peeling paint, water damage), and household crowding, due they create favorable habitats for pests [52]. According to our results, construction material is a relevant factor to determine the presence of cockroaches (Table 1). We suggest that some type of material may facilitate (i.e., metal sheet roof type) the entry of animals such as cockroaches. It is worth noting that these insects are omnivorous and resilient, and even under certain conditions may ingest various types of material, including plastic [53]. In Guatemala, construction materials were reported to be associated with the presence of other insects such as ticks (*Triatoma* spp.), where gypsum wall type, low floors, and tile roofs were risk factors for infestation [54]. Poor household conditions could determine both the presence and abundance of these insects such as cockroaches.

Studies report that in multi-story buildings, upper floors have been reported to have lower cockroach infestation rates compared to lower floors [49]. Also, different to our result (Table 1), vector control efforts are aligning with studies showing that this action can significantly reduce cockroach density in residential areas [8,55]. Nevertheless, these factors are heavily influenced by socioeconomic status, which can limit the effectiveness of control efforts [8,55].

The presence of domestic animals (i.e., presence of cats) has been reported to be associated with the presence of pests (e.g., *Mus* sp. and *Rattus* sp.) [54,56,57]. Following our results, the presence of cats increased the number of cockroaches (Table 1). We hypothesize that domestic animals, with their feces, exacerbate poor hygiene conditions in places where they are already limited, and cockroaches are attracted to these conditions. That is, many cockroaches, such as *P. americana*, that exhibit coprophagy behavior feed on vertebrate feces [2,3]. Therefore, the presence of feces from different animals would represent a food opportunity for cockroaches, more than just the presence of animals; however, this is a hypothesis that should be evaluated in future studies.

Inside households, cockroaches were mostly captured in kitchens, bedrooms, and backyards. The highest prevalence of parasitized cockroaches was found in sewers, bedrooms and kitchens. Our captures and prevalences of parasitism are aligned with reports in Nigeria, where the highest number of captured and parasitized roaches occurred in bathrooms, kitchens, and living rooms [58]. Other studies suggest that the presence of cockroaches in kitchens responds to the availability of food [7,8]. Occurrence of cockroaches in yards is probably due to the fact that they are open spaces with easy access for pests. While some trends in parasite prevalence were observed across capture sites, these should be interpreted with caution given the structural characteristics of rural homes. Many households lacked defined or separated rooms, and cockroaches could move freely within the living space [5]. Thus, site-specific differences may reflect activity patterns or environmental conditions rather than true spatial separation.

It is important to note that the abundance of insects can also be influenced by environmental factors such as temperature and humidity [20,35]. In temperate regions, cockroaches depend on warm environments, but in warmer regions they can live in natural areas [2,3]. We did not analyze the influence of environmental factors on the presence of cockroaches, so we recommend analyzing them in future studies about cockroaches in Guatemala.

Cockroaches have the ability to quickly move from one area to another within the same house throughout the day [5]. This behavior is significant from an epidemiological perspective, since it suggests that cockroaches could move back and forth, transporting a variety of microorganisms from outside to inside the house [3,59]. Additionally, educating residents on the importance of cockroach infestations may be an important strategy to reduce cockroach infestations and cockroach-borne diseases [8].

### Circulation and prevention of helminths parasites in cockroaches

In our study, we identified six helminth parasites and one acanthocephalan in *Periplaneta americana*. Five of the identified helminths, with the exception of *M. moniliformis*, belong to the order Oxyuroidea, family Thelastomatidae. Thelastomatids are classified as primary hosts, meaning they have no pathological effect on their cockroach hosts [9]. These helminths have already been identified in *P. americana* in countries across North and South America (e.g., USA, Peru), Europe (e.g., Germany, Bulgaria), and Asia (e.g., China, India) [16,39–41,55]. This is the first report of these helminth parasites in *P. americana* form Guatemala or any other Central American country.

Thelastomatids are reported as parasites or commensals of saprophytic terrestrial arthropods [39]. These have a cosmopolitan distribution, commonly found in association with the large intestine of *P. americana*, and they are considered non-pathogenic to humans [39,41,60]. Several species of both genera *Hammerschmidtiella* and *Leidynema* are commonly found co-infecting *P. americana* and other cockroach species, such as *Periplaneta australiasiae* and *Blatta orientalis* [41]. Both of them are not considered public health concerns. Nevertheless, their eggs closely resemble those of *Enterobius vermicularis*, a reported human parasite, so misidentification could lead to diagnostic confusion and underreporting [6,40]. Additionally, *Thelastoma* sp. has been reported co-infecting with *Hammerschmidtiella* [42] and in other arthropods living in ecologically similar habitats [61]. Co-infections of Thelastomatids helminths in cockroaches are commonly observed, with reports of up to 15 species co-infecting a single cockroach [9,42]. Also, cockroaches may accidentally ingest Thelastomoatoid eggs during coprophagy, because female Thelastomatoid nematodes lay eggs in the cockroach feces, which are passed in the cockroach feces [1,9].

The acanthocephalan *Moniliformis moniliformis* have been diagnosed both naturally and experimentally [15,16,18,62,63]. It depends on an intermediate host as *P. americana* and of a definitive host, the rodents, to finish their cycle life [63,64]*.* In cockroaches, *Moniliformis* larvae pass through the intestinal wall, and some can embed themselves in adipose tissue; this can harm the insect to varying degrees, depending on the extent of the infection [9]. *Moniliformis* is considered as a secondary parasite in cockroaches, meaning it could cause some damage to the host’s intestinal tract [9].

*Moniliformis moniliformis* has been reported in rodents in various countries and in human populations [65–67]. It is considered a parasite of public health interest due to its zoonotic nature, causing acanthocephaliasis [64,68,69]. In Guatemala, *M. moniliformis* has been identified in a *Rattus norvegicus* and accidentally in the necropsy of a dog [24], and in the human population, to date, it has not been diagnosed. It is important to consider the presence of *Moniliformis* in other animals, such as dogs, in future studies to better understand the role of other species in transmission or how they might be affected. However, the detection of the larval stage of *Moniliformis* in cockroaches indicates its circulation in the definitive host, particularly rodents, and in the study place [15,18,64].

Although the exact distribution of *Moniliformis moniliformis* is unknown, it probably occurs wherever rodents can be found [64,70]. In Latin America it has been reported in rodents (e.g., Peru, Chile) [71,72] and in human populations in some countries (e.g., Belize, Colombia) [70]. The majority of cases occur in children through ingestion of cockroaches [69,70]. This is very important because cockroaches, particularly *P. america*, which have a cosmopolitan distribution, allow them to be in close contact with the human population [3]. Human infection and clinical symptoms caused by acanthocephalans are rare, which likely makes proper detection and identification of these organisms difficult [64,70].

In general, Cockroaches serve as intermediate hosts or mechanical means to host different parasites [1–3,9]. Cockroaches are omnivorous insects, where young (nymphs) and adults share the same habitat and diet [3,9]. Their behavior allows them to remain and/or acquire the same microorganisms in the same ways (e.g., food, feces, contaminated materials) [3,9]. Experimental studies report that transmission of helminth parasites to cockroaches is usually direct, primarily through food [9].

The lack of effect of parasite prevalence and cockroach traits mirror the discoverers of [16] and [50], where no effect was observed from cockroach sex and age over parasite prevalence. However, parasite presence was higher in nymphs and females than in males, and in youth than in adults (Table 2). There are several factors that can affect parasitism by sex, such as diet, immunological response, hormone levels and body size [35,73–75]. Several studies have reported higher parasitism in female cockroaches [75,76]. Female cockroaches typically feed more and have longer lifespans than males, which could increase their cumulative exposure to environments and enhance the likelihood of parasitic infection [3]. Furthermore, reproductive females often exhibit reduced mobility while carrying egg cases (i.e., oothecae), leading to prolonged contact with microbe-saturated environments [3,77]. This reduced movement may also limit grooming behaviors that could otherwise help mitigate parasitic load [3,77].

Similar to our results, it has been reported that adults are more parasitized than nymphs [35]. However, in general, cockroach stage has not been found to affect parasite infection. As hosts develop, they undergo a variety of changes that can affect parasite infections [78]. Cockroaches live in gregarious, mixed-family herds in which nymphs and adults forage and rest together in the same microhabitats [3,10]. Therefore, they do not exhibit movement and diet differences that alter their exposure to infections. Physiological changes (e.g., insect immune responsiveness change with age) that accompany maturity could also affect parasitic loads [79]. Several biological, ecological, and behavioral factors may contribute to parasite presence in cockroaches.

Furthermore, the six genera of protozoa detected in *P. americana* in the present study, are of considerable pose both animal and human health risks due to their association with gastrointestinal disorders [6,20,58,80]. In Guatemala, however, only bacteria, specifically enterobacteria such as *Citrobacter freundii* and *Proteus mirabilis*, have been reported in cockroaches captured in hospitals [48], and protozoa such as *Toxoplasma gondii* have been documented in cockroaches collected from Guatemalan marketplaces [14]. It is recommendable that future studies incorporate protozoan analyses to provide a more comprehensive assessment of cockroaches as vectors of infectious agents.

## Limitations

Future studies should aim to include a larger sample size and explicitly compare different settings (e.g., such as rural and urban areas) and different cockroach species to better understand the ecological mechanisms influencing parasite prevalence. This study reflects only the distribution and infestation dynamics of *P. americana* due to the low number of *B. germanica* and *B. orientalis*. Although a generalized linear mixed model was used to assess the relationship between structural variables and cockroach abundance, some parameter estimates presented large standard errors. This is likely due to the relatively small sample size (70 households) and the number of explanatory variables included. As a result, the statistical power of the model to detect significant effects may be limited. These findings should therefore be interpreted with caution, and future studies with larger sample sizes are recommended to validate the observed trends.

## Conclusion

This study highlights the critical relationship between household factors with the presence of *P. americana* cockroaches and their helminth parasites in a rural Guatemalan community, providing a model of how similar risks can be addressed in other developing regions.

The detection of zoonotic parasites like *Moniliformis moniliformis* underscores the broader public health implications of cockroach infestations in countries where hygiene and infrastructure challenges are prevalent. Improving housing construction materials and implementing effective pest management measures could substantially reduce human exposure to these pests and their associated pathogens. Public health strategies targeting cockroach-borne pathogens should use pest control complemented with infrastructure improvement.

## Supporting information

S1 Table Data collection of household material, domestic animal and cockroaches presence in a rural community in Guatemala. (XLSX)

S2 Table Data collection of cockroaches captured in a rural community in Guatemala. (XLSX)

S3 Table Collinearity analysis of factors associated with cockroach abundance in a rural community in Guatemala. (XLSX)

S4 Table Analysis of contribution of each factor associated with the abundance of cockroaches in a rural community in Guatemala. Analysis according to AIC and Delta AIC (ΔAIC) represent the decrease in the Akaike information criterion (AIC) for the full model without that variable.
(XLSX)

S5 Table Diagnostic morphological traits used for the identification of helminth parasites collected from *Periplaneta americana*, summarizing the main distinguishing characters, life stages, and corresponding references. (XLSX)

S6 Table Prevalence and confidence interval by helminth parasites found in cockroaches (*Periplaneta americana*) in a rural community in Guatemala. (XLSX)
